# Chronic Stress, C-Reactive Protein, and Cognition Among Racially and Ethnically Diverse Older Adults

**DOI:** 10.1093/geroni/igaa057.1165

**Published:** 2020-12-16

**Authors:** Emily Morris, Laura Zahodne

**Affiliations:** University of Michigan, Ann Arbor, Michigan, United States

## Abstract

Objective: Previous research suggests that chronic stress is associated with worse cognitive aging, but minimal research has examined potential mechanisms and moderators of these associations. Chronic stress is known to increase inflammation (e.g., C-reactive protein [CRP]), which has in turn been associated with worse cognition among older adults. The present study examined whether (1) CRP mediates associations between chronic stress and episodic memory and verbal fluency; and (2) these relationships differ by race/ethnicity. Methods: Participants included 18,968 adults (64% non-Hispanic White; 19% non-Hispanic Black; 14% Hispanic; 3% non-Hispanic other race/ethnicity; Mage=71.8; SDage=6.0) from the Health and Retirement Study. Chronic stress was operationalized as the occurrence and impact of eight ongoing stressors. Cross-sectional, stratified mediation models were conducted for three cognitive outcomes: immediate recall, delayed recall, and verbal fluency. Covariates included sociodemographics and vascular disease burden. Results: Chronic stress was associated worse immediate recall (beta=-.028). Higher CRP was not associated with any cognitive domains. Non-Hispanic Black participants reported more chronic stress than non-Hispanic White and Hispanic participants. Chronic stress was less strongly associated with higher CRP in non-Hispanic Black (beta=-.035) participants than non-Hispanic White (beta=.046) or Hispanic (beta=.059) participants. Discussion: Chronic stress may negatively influence episodic memory, but findings do not suggest that CRP mediates links between chronic stress and cognition. CRP may not track as closely with chronic stress among non-Hispanic Black older adults who may experience additional risk factors for inflammation and/or adapt to increased chronic stress.

